# Adenosine Deaminase Acting on RNA (ADAR) Enzymes: A Journey from Weird to Wondrous

**DOI:** 10.1021/acs.accounts.3c00433

**Published:** 2023-10-31

**Authors:** Liam P. Keegan, Khadija Hajji, Mary A. O’Connell

**Affiliations:** CEITEC, Masaryk University, Kamenice 735/5, E35, Brno 62500, Czechia

## Abstract

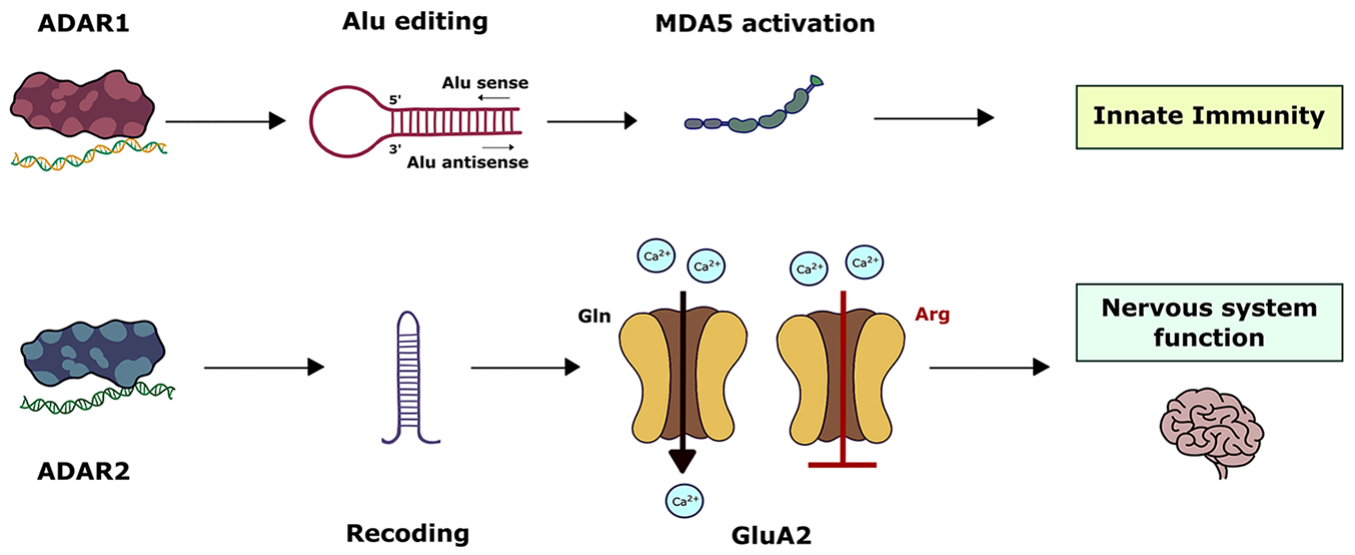

The adenosine deaminase acting on RNA (ADAR) enzymes that catalyze the conversion of adenosine to inosine in double-stranded (ds)RNA are evolutionarily conserved and are essential for many biological functions including nervous system function, hematopoiesis, and innate immunity. Initially it was assumed that the wide-ranging biological roles of ADARs are due to inosine in mRNA being read as guanosine by the translational machinery, allowing incomplete RNA editing in a target codon to generate two different proteins from the same primary transcript. In humans, there are approximately seventy-six positions that undergo site-specific editing in tissues at greater than 20% efficiency that result in recoding. Many of these transcripts are expressed in the central nervous system (CNS) and edited by ADAR2. Exploiting mouse genetic models revealed that transgenic mice lacking the gene encoding Adar2 die within 3 weeks of birth. Therefore, the role of ADAR2 in generating protein diversity in the nervous system is clear, but why is ADAR RNA editing activity essential in other biological processes, particularly editing mainly involving ADAR1? ADAR1 edits human transcripts having embedded Alu element inverted repeats (AluIRs), but the link from this activity to innate immunity activation was elusive. Mice lacking the gene encoding Adar1 are embryonically lethal, and a major breakthrough was the discovery that the role of Adar1 in innate immunity is due to its ability to edit such repetitive element inverted repeats which have the ability to form dsRNA in transcripts. The presence of inosine prevents activation of the dsRNA sensor melanoma differentiation-associated protein 5 (Mda5). Thus, inosine helps the cell discriminate self from non-self RNA, acting like a barcode on mRNA. As innate immunity is key to many different biological processes, the basis for this widespread biological role of the ADAR1 enzyme became evident.

Our group has been studying ADARs from the outset of research on these enzymes. In this Account, we give a historical perspective, moving from the initial purification of ADAR1 and ADAR2 and cloning of their encoding genes up to the current research focus in the field and what questions still remain to be addressed. We discuss the characterizations of the proteins, their localizations, posttranslational modifications, and dimerization, and how all of these affect their biological activities. Another aspect we explore is the use of mouse and *Drosophila* genetic models to study ADAR functions and how these were crucial in determining the biological functions of the ADAR proteins. Finally, we describe the severe consequences of rare mutations found in the human genes encoding ADAR1 and ADAR2.

## Key References

- GalloA.; KeeganL. P.; RingG. M.; O’ConnellM. A.An ADAR that edits transcripts encoding ion channel subunits functions as a dimer. EMBO J.2003, 22, 3421–34301284000410.1093/emboj/cdg327PMC165651.^1^*For catalytic activity, Drosophila Adar protein has to bind to dsRNA as a dimer*.
- HealeB. S.; KeeganL. P.; McGurkL.; MichlewskiG.; BrindleJ.; StantonC. M.; CaceresJ. F.; O’ConnellM. A.Editing independent effects of ADARs on the miRNA/siRNA pathways. EMBO J.2009, 28, 3145–31561971393210.1038/emboj.2009.244PMC2735678.^2^*ADAR enzymes have editing independent functions as they can bind RNA having dsRNA regions and inhibit other RNA processing events*.
- MannionN. M.; GreenwoodS. M.; YoungR.; CoxS.; BrindleJ.; ReadD.; NellåkerC.; VeselyC.; PontingC. P.; McLaughlinP. J.; JantschM. F.; DorinJ.; AdamsI. R.; ScaddenA. D. J.; ÖhmanM.; KeeganL. P.; O’ConnellM. A.The RNA-Editing Enzyme ADAR1 Controls Innate Immune Responses to RNA. Cell Reports2014, 9, 1482–14942545613710.1016/j.celrep.2014.10.041PMC4542304.^3^*Adar null mutant in mice can be rescued to allow live birth by combining this mutation with one in Mavs as this prevents downstream signaling via the RLR pathway*.
- DengP.; KhanA.; JacobsonD.; SambraniN.; McGurkL.; LiX.; JayasreeA.; HejatkoJ.; Shohat-OphirG.; O’ConnellM. A.; LiJ. B.; KeeganL. P.Adar RNA editing-dependent and -independent effects are required for brain and innate immune functions in *Drosophila*. Nat. Commun.2020, 11, 15803222128610.1038/s41467-020-15435-1PMC7101428.^4^*Despite Drosphila having a very divergent innate immune pathway, the role of Adar in innate immunity is evolutionarily conserved*.

## Background

With the advent of new technologies, the world of RNA modifications has been gradually revealed. Currently, there are approximately 170 different RNA modifications known in RNA in the three domains of life.^5^ Most RNA modifications occur in tRNAs; however, recent excitement in this research field has been generated by the occurrence of RNA modifications in mRNA. Deamination of adenosine to inosine is among the most abundant modifications in mRNA with over a hundred million sites in the human genome.^6^ The ADAR enzymes that catalyze adenosine deamination in dsRNA recognize the A-form structure and have little sequence specificity, with base preferences limited to the edited adenosine and the bases on either side of it. Inosine in tRNA anticodons is generated by the adenosine deaminase acting on tRNA (ADAT) enzymes, whose catalytic domains are smaller than those of ADARs but with similar deaminase active sites.^7^

Unlike *N*^6^-methyladenosine (m^6^A) modification, there are no epigenetic erasers for inosine; also, there are no particular classes of readers that bind to it and perform a particular function such as increasing or decreasing the stability of inosine containing mRNA. Instead, inosine itself within dsRNA is a signal for the innate immune sensors that the RNA is “self” and to not initiate an immune response (for review, see ref (8)); it could be argued that the innate immune dsRNA sensors are the inosine readers. A screen to identify proteins that bind to dsRNA oligos containing inosine identified proteins that localize to stress granules,^9^ where ADAR1 is also located.

Another difference between inosine and m^6^A modification is how it is identified. m^6^A requires identification and immunoprecipitation first by m^6^A antibodies followed by RNA sequencing, miCLIP or meRIP (for review, see ref (10)). This can be problematic if the anti-m^6^A RNA antibodies are not highly m^6^A-specific. On the other hand, inosine base pairs with cytosine resulting in the incorporation of guanosine at the editing site by reverse transcriptase when cDNA is generated from edited RNA. Therefore, it is easy to identify with confidence new editing positions as adenosine in the genomic DNA appears as guanosine in the cDNA; usually there is a mixed A/G peak in the sequence. Inosine can be translated as guanosine by the translation machinery,^11^ so if RNA editing occurs within the coding regions, then another amino acid can be incorporated at the edited position. This editing event is called site-specific, and if it occurs within an open reading frame, it may be a recoding RNA editing event that can impact the function of the encoded protein. ADAR2 is mainly responsible for this recoding editing,^12^ whereas ADAR1 edits transcripts having embedded repetitive element inverted repeats, such as Alu elements, that can form AluIR dsRNA (for review, see ref (13)). Intriguingly, ADAR1 editing activity is essential to limit innate immune responses even though AluIR edits occur with minimal site specificity and only at very low efficiencies.^6^

In this review, we will focus on the contribution our group has made to the field of RNA editing over the past 30 years.

## Purification and Cloning of ADARs

When performing experiments with antisense RNA in *Xenopus* oocytes, a strand-separating apparent unwinding activity was detected.^14,15^ Further investigation showed that this activity did not unwind dsRNA but instead converted adenosine to inosine so that it no longer base-paired well with uracil, allowing edited dsRNA strands to separate.^16^ When the initial discovery of this modification was made, it was noted that in some viral infections there was a conversion of adenosine to guanosine in the sequences of some cDNAs encoding viral proteins after prolonged virus infection.^17^ This is the hallmark of RNA editing, and thus, it was presumed that the same editing activity was responsible. Therefore, the quest began to purify the enzyme responsible and to clone the encoding gene.^18−20^

**Figure 1:**
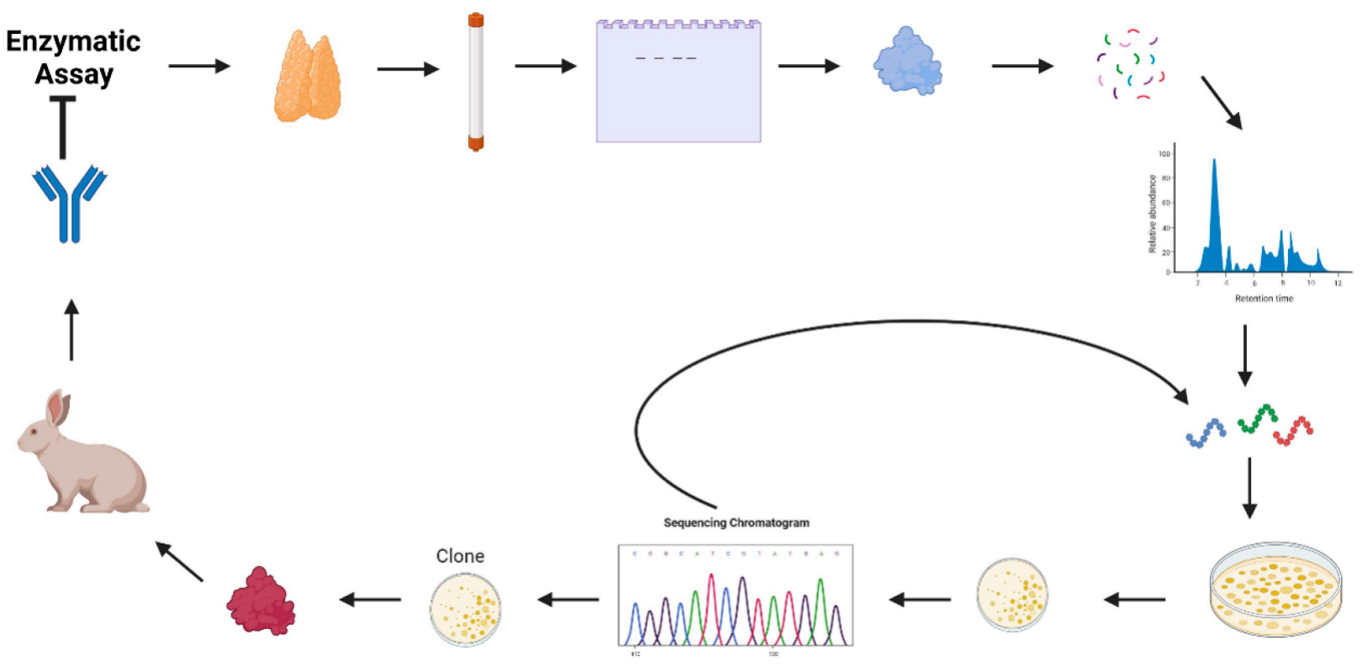
Purification of ADAR1 and cloning of the *ADAR* gene. To purify an unknown enzyme, the first requirement is a specific activity assay for that enzyme. The assay for ADAR1 was the conversion of adenosine into inosine in dsRNA. The dsRNA was *in vitro* transcribed, labeled with radioactive α-ATP, and annealed. After incubation with extract containing ADAR1, the dsRNA was digested, and the products were separated by thin layer chromatography (TLC) as the migration of inosine is faster than that of adenosine. ADAR1 was purified to homogeneity >340,000-fold from 7 kg of calf thymus over 6 columns. The protein was digested and the peptides were analyzed by LC/MS to obtain peptide sequences. Radio-labelled oligonucleotides with redundant codon sequences were used to screen a human cDNA library. Any positive clones were rescreened and sequenced. Eventually a full-length cDNA clone was obtained. A part of the open reading frame was overexpressed in *E. coli* and the recombinant protein was used as an antigen to generate polyclonal antibodies in rabbits. This antibody was then used in the dsRNA adenosine deamination activity assay to demonstrate that it specifically inhibited this enzymatic activity. This was the proof that the ADAR1 protein was purified and cloned.

As there was very little cDNA sequence data available in the early 1990s, one first had to purify the enzymatic activity to homogeneity from abundant inexpensive sources such as calf thymus and then scale up the purification to generate a sufficient amount of protein so that peptide sequences could be obtained by mass spectrometry (Figure 1). Redundant oligos corresponding to the peptide sequence were synthesized and used to screen cDNA libraries by oligonucleotide hybridization. Once a positive cDNA clone was obtained it was sequenced. Usually, the cDNA clone only contained a part of the coding sequence of interest so multiple rounds of library screening had to be performed to obtain a full-length cDNA clone. A part of this novel open reading frame was then cloned to overexpress a protein domain in *E. coli* to use as an antigen for injection into rabbits to generate polyclonal antibodies. These antibodies were tested for their capacity to inhibit the enzymatic activity that was used for the purification of the protein; it was only at this stage that one knew if one was successful in purifying the original enzyme and cloning the correct gene. This scheme was used to purify and clone mammalian ADAR1.^20,21^ There are two isoforms of the protein, a predominantly nuclear isoform (p110) and a cytoplasmic interferon (IFN) inducible isoform that has an extended amino-terminus (p150).^22^

The first RNA editing site identified was the critical *GRIA2* Q/R site, where a glutamine residue is converted into an arginine in the mammalian *GRIA2* transcript encoding the glutamate 2 (GluA2) subunit of α-amino-3-hydroxy-5-methyl-4-isoxazolepropionic acid (AMPA) receptors.^23^ RNA editing is essential at this position as it regulates the calcium permeability of AMPA receptors; heterotetrameric channels containing a GluA2 subunit with glutamine in the pore are permeable to calcium, whereas those with arginine are not. This position is edited to almost 100% and when it does not occur, there is an influx of calcium resulting in seizures and neuronal cell death.

It was presumed that ADAR1 would catalyze the editing at the *GRIA2* Q/R position, and when we did not observe it with purified ADAR1, we assumed that a specificity factor was lacking that bound to ADAR1. In an attempt to characterize this specificity factor, a sizing column was used to estimate the molecular weight of the complex required to edit the *GRIA2* Q/R editing site. Surprisingly, the adenosine deaminase activity that edited the Q/R site had a smaller molecular weight than ADAR1, and thus there was another RNA editing enzyme: ADAR2.^24^

A scheme similar to that described above was used for ADAR2. Whereas ADAR1 was purified from 7 kg of calf thymus, ADAR2 was purified from 14.67 g of HeLa nuclear extract, which is the equivalent of approximately 1500 L of HeLa cells.^25^ We used HeLa nuclear extracts rather than calf thymus as we were aware that the second ADAR activity was enriched in it.^24^ Whereas purified ADAR1 was stable during purification and subsequent studies on the enzymatic activity, ADAR2 was extremely labile and very sensitive to freeze–thawing. Subsequently the *ADARB1* gene encoding ADAR2 was cloned, and it was demonstrated that it is responsible for editing the Q/R site in the *GRIA2* transcript;^26,27^ the recombinant ADAR2 no longer showed the lability of endogenous ADAR2, which might be due to posttranslational modifications present only on the endogenous protein.

## Characterization of the ADAR Enzymes

A key question was how ADAR enzymes recognize their target transcripts. It was demonstrated that ADAR2 had stable binding at edited sites whereas only transient binding occurred at nonselective, promiscuous editing sites.^28^ Therefore, we reasoned that perhaps the ADAR enzymes formed dimers similar to other members of their family of cytidine deaminases (CDAs) and ADATs. We presumed that this stable binding was required for ADAR deamination activity at selected sites and that the ADAR enzymes scanned dsRNA for these specific sites. This turned out to be true as we demonstrated that *Drosophila* Adar protein indeed requires dimerization on dsRNA for enzymatic activity.^1^ This result was in contrast to publications from two other groups, one stating that ADAR2 dsRNA binding domains (dsRBDs) interact and that ADAR2 did not form dimers.^29^ The other publication stated that ADAR proteins formed dimers but does not require binding to dsRNA for their formation.^30^ This controversy surrounding ADAR dimerization was finally resolved by the structure of an ADAR2–dsRNA cocrystal having a truncated ADAR2 composed of the deaminase domain and dsRBDII binding to an edited dsRNA substrate. Indeed, ADAR2 forms dimers on dsRNA, and surprisingly, they are asymmetric; one deaminase domain uses its active site and dsRNA-binding face to bind to the back of another deaminase domain, which performs the editing.^31^ This was in contrast to our findings that *Drosophila* Adar,^1^ which is an orthologue of ADAR2, requires its amino terminus for dimer formation. As currently there is no structure for the full length ADAR2 protein, it is possible that the amino terminus and perhaps dsRBD1 are also involved in dimerization. Another possibility is that the result is an artifact of the yeast two-hybrid system used to demonstrate the involvement of the amino terminus of *Drosophila* Adar in dimerization.

As rabbit polyclonal antibodies were generated in the process of cloning the genes encoding ADAR1 and ADAR2, they were used to characterize the localizations of both endogenous and overexpressed ADAR proteins.^32^ When ADAR1p150 was expressed from transfected plasmids in cells, it generated both p110 and p150 isoforms due to the use of alternative starting methionines. When the M296 AUG for p110 was mutated, the ribosome scanned downstream to the next in-frame methionine at M337.

As previously reported, ADAR1 p110 is mainly nuclear and ADAR1 p150 is mainly cytoplasmic,^22^ whereas ADAR2 is only present in the nucleus.^32^ However, when ADAR1 p110 or ADAR2 is overexpressed then both nuclear proteins are in constant flux in and out of the nucleolus.^32,33^ When a plasmid encoding an editing-competent substrate was transfected into the cells, then these ADAR proteins relocalized from the nucleolus to a nuclear site where the substrate was being transcribed; this did not occur if the plasmid encoded an RNA that did not form dsRNA. Thus, the nucleolus stores excess ADAR proteins, which is not surprising, as the nucleolus contains high levels of Alu RNAs.^34^ The ADAR proteins can relocalize from the nucleolus and be recruited to a site for RNA editing when required.

## Posttranslational Regulation of ADAR Enzymes

**Figure 2:**
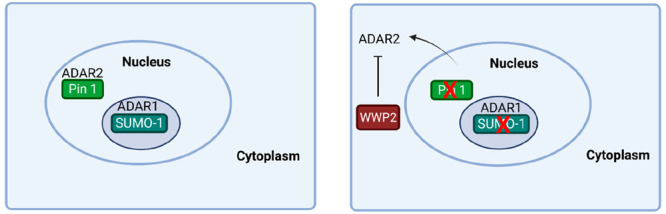
Posttranslational modifications can affect ADAR localization. ADAR1 is modified by SUMO and is localized to the nucleolus. When ADAR1 is mutated to prevent this modification, it does not alter the localization of ADAR1 to the nucleolus. ADAR2 is phosphorylated by an unknown kinase and is then a substrate for Pin1 that catalyzes the cis/trans isomerization of the peptide bond at proline close to the amino terminus. This modification contributes to the nuclear retention of ADAR2; in its absence ADAR2 can localize to the cytoplasm where it is a substrate for WWP2.

ADAR1 is modified by small ubiquitin-like modifier-1 (SUMO-1), on lysine 418^35^ (Figure 2). This modification is very dynamic and reversible and can have various consequences for target proteins; it changes substrate interactions such as DNA or RNA interactions with the modified protein, or it affects enzymatic activities. A mutation in ADAR1 at position K418 was generated that prevented the conjugation of SUMO-1 to ADAR1, and this led to an increase in RNA editing activity *in vitro*.^35^ It is interesting that many proteins involved in the innate immune response undergo modification by SUMO-1.^36^ ADAR1 co-localizes with SUMO-1 in the nucleolus; however, SUMO-1 is not required for this localization as the mutant that is not SUMOylated at K418 also localizes there.

ADAR2 is regulated by the phosphorylation-dependent peptidyl-prolyl cis/trans isomerase Pin1 (peptidyl-prolyl isomerase NIMA interacting protein 1).^37^ Pin1 binds to a consensus sequence (which is a phosphorylated serine/threonine residue preceding a proline) and catalyzes the cis/trans isomerization of the peptide bond at proline. Pin1 binds to the amino-terminal region of ADAR2 and in its absence ADAR2 mis-localizes to the cytoplasm (Figure 2). There it is degraded by the HECT E3 ubiquitin ligase, WWP2. This E3 ligase was identified as one of the proteins that co-purified with ADAR2 when it was originally purified.^25^ WWP2 binds to a PPxY motif, and this is present twice in ADAR2. When both of these motifs are mutated, ADAR2 is more stable and not ubiquitinated in the cytoplasm. Therefore, there is a regulatory mechanism to accumulate ADAR2 in the nucleus; it has a nuclear localization sequence,^38^ and it is phosphorylated and undergoes a cis/trans isomerization by Pin1. If Pin1 is absent and some ADAR2 is present in the cytoplasm, then it is degraded by WWP2.^37^ Thus, the carefully maintained absence of ADAR2 in the cytoplasm leaves ADAR1 p150 as the sole editing enzyme to control the level of A–I editing in the cytoplasm in response to IFN induction. ADAR2 can be relocalized to the cytoplasm if a deletion is made at the amino terminus,^39^ and when ADAR2 Δ4–72 is expressed from a plasmid transfected into mouse embryonic fibroblasts (MEFs) lacking *Adar* (the gene encoding mouse Adar1), ADAR2 Δ4–72 is active in the cytoplasm and compensates for the lack of the mouse Adar1 protein.^3^ ADAR2 Δ4–72 is stable in the cytoplasm as it has only one copy of the PPxY motif and two are required for its degradation by WWP2.^38^

## RNA Editing—Independent Effects of ADARs

The ADAR proteins are dsRNA binding proteins in addition to being RNA editing enzymes. Therefore, ADARs could have biological functions that are independent of RNA editing. This hypothesis was supported by the observation that ADAR3 that is expressed in the brain is enzymatically inactive^40^ as are the two ADAD proteins (adenosine deaminase domain containing proteins 1 and 2) that are expressed in the testis and are homologous to ADAR proteins.^41^ So, in mammals, there are two active enzymes and three inactive paralogues that are all dsRNA binding proteins in this extended protein family.

We chose to study the properties of ADAR proteins that are important for micro-RNA (miRNA) processing; specifically, processing of the *miR-376* cluster *in vitro*,^2^ as it was previously demonstrated that RNA editing by ADARs could retarget miRNAs of this cluster.^42^ We demonstrated that inactive ADAR2 could bind pri-*mir-376a2* and inhibit Drosha cleavage. In the *Drosophila* model, the expression of inactive human ADAR1 also inhibited the siRNA pathway. Thus, by binding to dsRNA, the ADAR proteins can antagonize other pathways, and this is independent of catalytic activity. This was the first demonstration of an editing-independent function of ADAR proteins.

## Mouse Genetic Models for Studying ADAR1

Mice that are null for *Adar* are embryonic lethal and do not survive beyond E12.5.^43,44^ They display an overproduction of IFN, liver disintegration, and loss of hematopoietic cells, as well as widespread apoptosis. For more than ten years, many groups sought Adar1-edited targets to rescue this lethal phenotype, as they presumed that it might be rescued by the edited isoform of its key target transcript. This would be similar to the rescue of the *Adarb1* (gene name for Adar2) by knocking-in the edited isoform (R) of *Gria2*,^45^ implying that in a mouse model the most important target of RNA editing by Adar2 is the *Gria2* transcript at the Q/R site.

**Figure 3:**
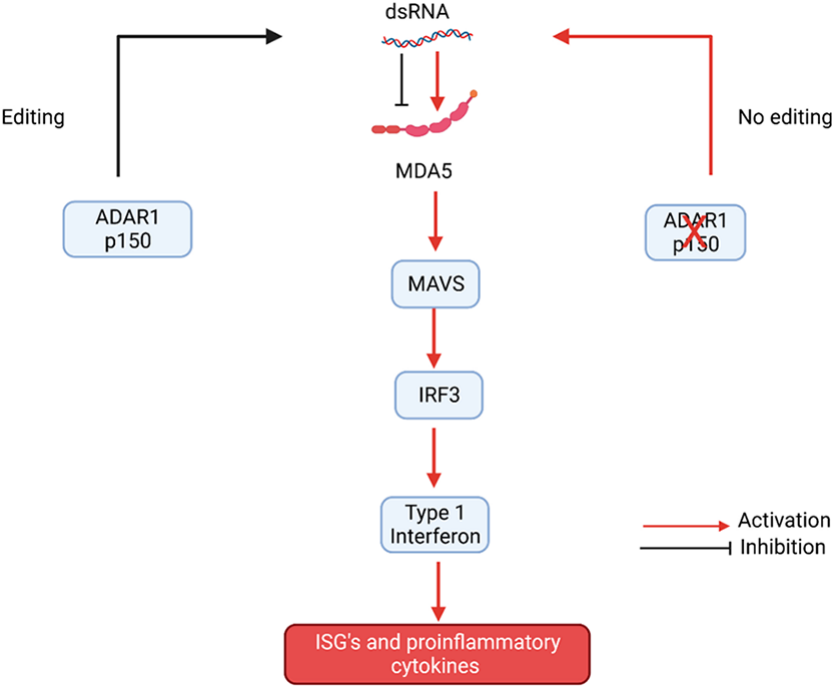
ADAR1 can edit dsRNA, and this prevents activation of the dsRNA sensor, MDA5. In the absence of ADAR1 or ADAR1 editing activity, dsRNA can bind to and activate MDA5, and the subsequent downstream proteins such as MAVS and IRF3. This results in the expression of Type 1 IFN and subsequent expression of ISGs and inflammatory cytokines.

However, no transcript or miRNA was identified in which editing by Adar1 was essential for mouse embryonic development. ADAR1 edits transcripts encoding short interspersed nuclear element (SINE) inverted repeats, such as AluIRs in humans (for review, see ref (13)). When two Alu elements are embedded in reverse orientations in a longer transcript, they can form duplex RNA that is targeted by ADAR1. We reasoned that editing of this repetitive element dsRNA was responsible for the increased innate immune induction in the *Adar* null mice. We took a genetic approach and crossed *Adar* null mice with mouse mutants lacking the mitochondrial antiviral-signaling protein (Mavs), which is downstream of two antiviral receptors for dsRNA, retinoic acid-inducible gene I (Rig-I) and melanoma differentiation-associated protein 5 (Mda5). The mutation removing Mavs blocks all downstream signaling for IFN induction and the *Adar Mavs* double mutant mice survive to live birth^3^ (Figure 3). Analysis of RNA Seq data revealed that the levels of IFN stimulated gene (ISG) transcripts and proinflammatory cytokine transcripts are reduced in *Adar Mavs* double mutants. Therefore, ADAR1 edits dsRNA, thus preventing it from activating the innate immune response. Another group also rescued the *Adar*^*E912A*^ catalytically inactive mutant phenotype by generating an *Adar*^*E912A*^*Ifih1* (the gene encoding Mda5)^46^ double mutant, demonstrating that Mda5 is the main dsRNA receptor that binds unedited RNA, and activating the downstream IFN response. Mouse embryos expressing inactive Adar1 E912A live about 2 days longer than *Adar* null mouse embryos, and when they are combined with *Ifih1*, they have a normal lifespan.^46^ This validated *in vivo* what we had previously reported, that ADARs have functions that are independent of RNA editing.^2^

*Adar Trp53* null MEFs were created, as *Adar* MEFs could not be cultured, and derivative stable cell lines were generated that expressed either ADAR1 p110 or ADAR1 p150, inactive ADAR1 or an ADAR2 N-terminal deletion that localizes to the cytoplasm.^3^ A robust immune response was elicited in *Adar Trp53* null MEFs by serum starvation, and derivative stable cell lines expressing active ADAR1 or cytoplasmic ADAR2 effectively suppressed the ISG inductions in this immune response, whereas enzymatically inactive ADAR1 was the least effective, though there was some effect. This result demonstrates that cytoplasmic ADAR2 is capable of reducing the innate immune response and that a significant reduction by ADAR1 requires editing activity.

An immune response was induced in *Adar Trp53* MEFs by transfecting them with an *in vitro* transcribed RNA^3^ which gives an immune response likely due to incomplete capping of the RNA.^47^ This immune induction was reduced by transfecting into the cells double-stranded oligos containing four inosine–uracil pairs in the middle. This demonstrates that the cell uses inosine in dsRNA to discriminate self from non-self RNA and to turn off innate immunity. It is possible that inosine dsRNA acts directly as an inhibitor of MDA5, preventing its oligomerization and activation despite the presence of other immunogenic dsRNAs in the cell. This hypothesis is contrary to the idea that RNA editing destabilizes and unwinds dsRNA in AluIRs; however, it has been demonstrated that RNA editing can stabilize dsRNA structures as inosine improves base paring (I:C) at many editing sites.^48^ Perhaps in the future, dsRNA oligos containing inosine or other modified nucleosides could be used to reduce chronic inflammation.

### *DrosophilaAdar*

**Figure 4:**
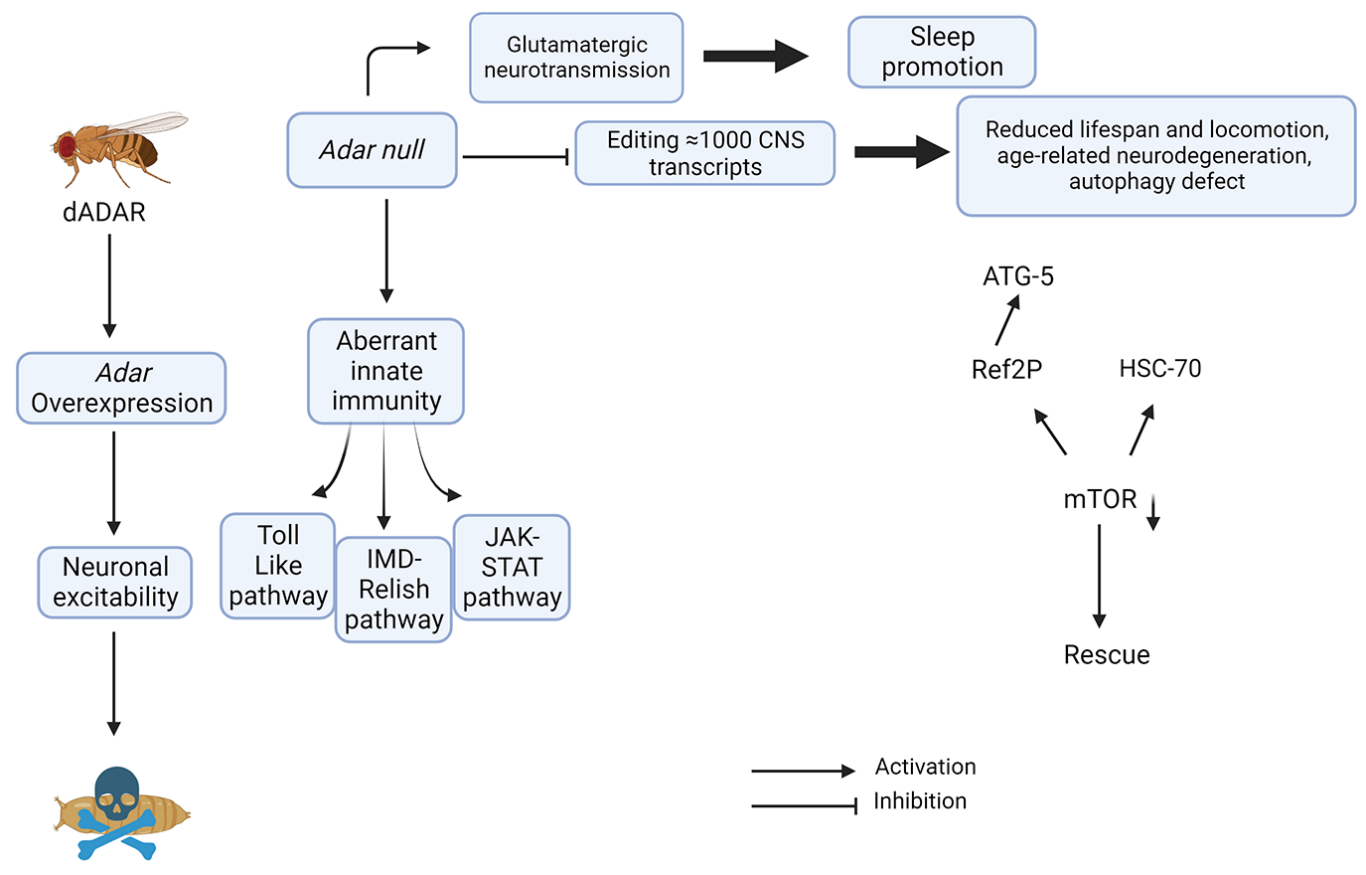
Biological roles of *Adar* in *Drosophila*. In *Drosophila*, there is one *Adar* gene and overexpression of it results in neuronal excitability and lethality. *Adar* null mutants are viable; they produce an aberrant innate immune response with activation of the Toll-like, IMD-Relish, and JAK-STAT pathways. *Adar* mutants have an increase in glutamatergic neurotransmission resulting in elevated sleep. *Adar* edits approximately over a 1000 recoding events within the CNS transcripts; the null mutant displays a reduced lifespan, problems with locomotion, and age related neurodegeneration due to defects in autophagy.

There is only one *Adar* gene in *Drosophila*,^49^ and it is an orthologue of human *ADARB1* (ADAR2).^50^ Mutant flies lacking Adar activity are obtained in reduced numbers from crosses because some of the mutants die as larvae, *Adar* mutant flies have locomotion defects and ataxia and suffer from age-related neurodegeneration^49^ (Figure 4). They accumulate large holes in their brain, particularly in the mushroom body, which is equivalent to the hippocampus in humans. This neurodegeneration is caused by insufficient canonical autophagy in the *Adar* mutant flies, and it involves an aberrant accumulation of synaptic vesicles on the axonal sides of synapses.^51^ Increasing canonical autophagy by a reduction in Tor kinase activity rescues the *Adar* null neurological phenotype. Endosomal microautophagy is another type of autophagy that is also involved in proteostasis at presynaptic active zones in *Drosophila*. It requires Hsc70-4 to bind to proteins having a KFERQ motif and targets them to endosomes for degradation. Increasing Hsc70-4, thereby increasing endosomal microautophagy, also reduces synaptic vehicles and suppresses the *Adar* mutant neurodegeneration phenotype.^51^ The *Adar* loss of function mutant *Drosophila* also has very much increased numbers of synaptic vesicles in the axonal boutons of larval motor neurons.^52^ A more recent study showed that *Adar* hypomorphic mutant flies have sleep defects which arise from excessive glutamatergic synaptic vesicles.^53^

Adar edits over 1300 specific positions, including about a thousand recoding sites, in approximately six hundred transcripts in *Drosophila*. Editing increases during development, with fewer sites and lower editing efficiencies in embryos and more sites and higher editing in adult flies; Adar levels are increased by ecdysone at metamorphosis.^54^*Drosophila* Adar protein is mainly expressed in the CNS, and it primarily edits transcripts expressed there.^49^*Adar* has an alternative splicing of an exon that is located between dsRBD1 and dsRBD2. This 3a exon is expressed in embryos and larva, whereas the adult 3/4 transcript lacks this exon.^55^ Interestingly the spacing between dsRBDs when exon 3a is included is similar to the spacing between dsRBDs in ADAR2, whereas without exon 3a the spacing resembles that in ADAR1.^56^ Surprisingly Adar edits its own *Adar* transcript to convert a serine to a glycine residue (S/G)^55,57^ near the deaminase active site. Most editing sites have editing site complementary sequences (ECSs) in the next downstream intron to form the editing substrate; however, the dsRNA required for RNA editing at the *Adar S*/*G* site is present within the coding exon so that it does not have to be edited co-transcriptionally before splicing.^58^ The level of this *Adar S*/*G* self-editing is low in embryos and rises to 40% in adult flies. The edited Adar G isoform is less active than the genome-encoded Adar S isoform, both *in vivo* and *in vitro*. Therefore, by regulating alternative splicing as well as by self-editing of the *Adar* transcript, *Drosophila* can generate different isoforms of proteins expressed in the nervous system that change enzymatic activity during development.

Overexpressing the active genome encoded Adar S isoform prematurely and at high levels in embryos and larvae is lethal as there is an increase of editing in embryos to levels more like those in adults.^57^ An unedited, genome-encoded Adar S isoform in the 3/4 splice isoform cDNA was generated in which the serine codon is changed to remove the adenosine that can be edited. *Gal4/UAS*-driven *Adar 3/4 S* overexpression is lethal in embryos and larvae, so it was placed under the control of Gal80^ts^ and a genetic screen was performed to identify suppressors of ADAR overexpression (Adar OE) lethality at higher temperatures where the Gal80^ts^ is inactive and *Gal4* drives *Adar 3/4 S* overexpression.^59^ One suppressor identified was the resistance to dieldrin (*Rdl*) gene, which encodes an inhibitory GABA-gated chloride channel. Electrophysiological examination of larval motor neuron axons showed that *Adar*^*5G1*^ loss of function mutants have increased excitability, whereas Adar OE motor neuron axons show decreased excitability which is corrected by halving the dosage of the *Rdl* gene under screening conditions.

Human ADAR2 expressed in *Adar* mutant *Drosophila* rescues all *Adar* mutant phenotypes tested and edits the range of *Drosophila* editing sites similarly to *Drosophila* Adar.^50^ Human ADAR1 expressed in *Adar* mutant *Drosophila* also edits many, but not all, of the fly editing sites but the order of site preference is different and ADAR1 does not rescue *Adar* mutant phenotypes. ADAR1 p150 and p110 are both toxic in *Drosophila* and affect RNAi and probably also miRNA processing.^2^

An inactive *Adar*^*E374A*^ mutant was generated by CRISPR/Cas9.^4^ This *Adar*^*E374A*^ mutant has a similar phenotype as the *Adar* null mutant, implying that Adar editing activity is required for locomotion and prevention of age-related neurodegeneration. However, when the inactive Adar E374A isoform was expressed from a *UAS-Adar*^*E374A*^ construct to approximately four times the normal physiological level, it rescued the neurodegenerative phenotype but not locomotion. This again is evidence that Adar protein has editing-independent effects.^2^

Surprisingly, the loss of Adar activity also leads to immune induction in *Drosophila*, despite the *Drosophila* enzyme being the ortholog of ADAR2 and not ADAR1.^4^ This suggests that the one *Drosophila* protein has characteristics of both mammalian ADAR1 and ADAR2 and may edit wider sets of substrates, some of which affect CNS while others affect immune function. Innate immunity in *Drosophila* is very different from that in mammals as it lacks IFN and the RIG-I like receptor (RLR) pathway; however, it is significant that the role of ADAR in immunity is evolutionarily conserved. The innate immune induction in *Adar* mutant *Drosophila* is suppressed by silencing Dicer-2, which is a dsRNA sensor in *Drosophila* that has a similar helicase domain to MDA5.

## Human Mutations in Genes Encoding ADARs

Human mutations in *ADAR* can cause Aicardi-Goutières syndrome (AGS6);^60^*ADAR* was the sixth gene identified in which human mutations cause this syndrome (Table 1). AGS results in inflammation and aberrant high IFN and ISG expression, particularly in the brain, and it presents in children with symptoms similar to those of a congenital virus infection with high levels of IFN. This can lead to calcification in the brain and can be fatal in children who die in early childhood. Mutations in *ADAR* that cause AGS are mostly biallelic and primarily located within the deaminase domain, although one is in the first Z RNA-binding domain. The mutant ADAR1 proteins in any patient generally led to only a moderate decrease in overall editing activity, as a mutation with a greater loss of function may be fatal, as such mutants are in mice. The AGS mutations in *ADAR* had a greater effect on RNA editing activity when they were expressed in the ADAR p150 isoform rather than in the p110 isoform.^3^ This suggested that the ADAR1 p150 isoform was more critical, and this hypothesis was validated later when it was demonstrated that the cytoplasmic ADAR1 P150 isoform was essential for the innate immune response suppression.^61^

**Table 1:** Diseases Associated with Mutations in the Human Genes Encoding ADAR1 and ADAR2

gene	ADAR associated diseases	type of mutation	refs
*ADAR*	Aicardi-Goutières Syndrome 6	Missense mutations in the catalytic domain and one in the Z-DNA binding domain	Rice et al.^60^
	Bilateral striatal necrosis	Missense mutations	Livingston et al.^70^
	Dyschromatosis Symmetrica Hereditaria	Missense mutation, Stop and frameshift mutations	Miyamura et al.^71^
*ADARB1*	Early infantile-onset seizures	Missense mutations, One deletion p.(Leu415PhefsTer14) causes frameshift and premature stop codon	Tan et al.;^64^ Maroofian et al.^63^

Mutations in *ADAR* can also cause Dyschromatosis Symmetrica Hereditaria 1 (DSH1), an autosomal dominant disorder with milder IFN induction and a childhood onset of hypo- and hyperpigmented macules on the extremities,^62^ which has been reported mainly in Japan and China. The mutations causing DSH are frameshifts and translation stops, leading to truncated ADAR1 proteins; however, DSH1 symptoms arise as a dominant phenotype and may be due to haploinsufficiency in human *ADAR* heterozygotes.

To date, seven biallelic human disease variants have been reported to be located in *ADARB1* (gene encoding ADAR2).^63,64^ These patients show severe developmental delay, microcephaly, and intellectual disability, and some have intractable early infantile-onset seizures (Table 1). ADAR2 is responsible for editing the Q/R position in the *GRIA2* transcript and mice lacking *Adarb1* have seizures and die with 3 weeks of birth.^45^ However, this mouse mutant phenotype is rescued by knocking in the edited version of *Gria2*^*R*^. Therefore, it is likely that the severe human condition is also due to the lack of RNA editing of the Q/R site in *GRIA2* by ADAR2. Similarly to ADAR1 AGS mutants, some of these individual mutations in *ADARB1* do not lead to a dramatic loss in ADAR2 activity in *in vitro* assays, as the most severe mutations affecting ADAR2 activity may be fatal earlier in development.

## Outlook

Research on RNA editing by ADARs has been an outlier for many years. It seemed inconceivable to many that these enzymes would dare to modify the genetic code within RNAs. ADARs were considered to be oddballs, “loose cannons within the cell”. However, what was obvious from early in this field was that these enzymes were highly evolutionarily conserved^65^ as well as being essential.^43−45^ This implied that their novel biological functions had not yet been recognized.

Currently the situation regarding research on ADARs is quite different. The role of ADAR2 in the brain is quite well established, and new roles of ADAR2 in human cardiovascular disease is a promising area of research.^66^ What is rather surprising is the high levels of RNA editing by ADAR2 in the vascular system, which are ten times higher than those in the brain. Cardiovascular defects were not observed in the *Adarb1 Gria2*^*R*^ mice; however, mice are not a perfect model for studying human cardiovascular disease,^67^ and it is possible that the role of ADAR2 in human heart disease is underestimated.

ADAR1’s role in innate immunity is now well established, and ADAR1 has also been shown to be an important therapeutic target in cancer treatment, in particular in those types of cancer that have an increased ISG transcript signature.^68^ A reduction in ADAR1 activity has been shown to augment the response to checkpoint inhibitor anti-PD-L1/PD-1 (programmed cell death ligand 1) antibodies^69^ as reduced RNA editing activates the RLR pathway and IFN response thus overcoming immunotherapy resistance. However, there are still many open questions.

One puzzling question is whether there are specific unedited transcripts that trigger the innate immune response in *Adar* mutants. ADAR1 edits AluIRs in transcripts; however, the level of this editing is low, often just one percent;^6^ therefore 99 percent of adenosines remain unedited, so it is unclear how this low level of editing can prevent activation of the innate immune response. Two scenarios could possibly explain this; highly duplex transcripts exist that have hyperediting, which is highly efficient editing of multiple adenosines in a short region to prevent it from being immunogenic. Hyperedited sites would likely be species-specific and are difficult to find in sequence data, and these have not yet been identified. Another possibility is that inosine in dsRNA prevents the activation of MDA5, locking it in an off position so that only a few inosine containing transcripts are required. Another major question is what are the editing independent functions of ADARs?

ADAR1 has a central and pivotal role in innate immunity. As ADAR1 controls the level of unedited cellular dsRNA, it regulates the homeostasis within the cell. Lowered levels of edited dsRNA will trigger an innate immune response, whereas high levels of edited dsRNA turn it off. Therefore, if we can regulate the level of RNA editing by ADAR1, we can regulate particular immune responses, increase them to fight an infection, and decrease them during chronic inflammation. This could also be game changing in relation to infectious diseases. Harvesting the therapeutic potential of these “oddball” enzymes will likely have major beneficial consequences.

## Abbreviations

ADAR: adenosine deaminase acting on RNA
ds: double-stranded
CNS: central nervous system
AluIR: Alu element inverted repeats
MDA5: melanoma differentiation-associated protein 5
ADAT: adenosine deaminase acting on tRNA
m^6^A: *N*^6^-methyladenosine
meRIP: methyl-RNA immunoprecipitation
miCLIP: mapping m^6^A at individual-nucleotide resolution using cross-linking and immunoprecipitation
IFN: interferon
GluA2: glutamate 2 subunit
AMPA: α-amino-3-hydroxy-5-methyl-4-isoxazolepropionic acid
CDA: cytidine deaminase
dsRBD: dsRNA binding domain
SUMO-1: small ubiquitin-like modifier-1
Pin1: peptidyl-prolyl isomerase NIMA interacting protein 1
MEF: mouse embryonic fibroblast
*ADAR*: gene encoding ADAR1
ADAD proteins: adenosine deaminase domain containing protein 1 and 2
miRNA: micro-RNA
MAVS: mitochondrial antiviral-signaling protein
Rig-I: retinoic acid-inducible gene I
ISG: IFN stimulated gene
*Ifih1*: the gene encoding MDA5
*Rdl*: Resistance to dieldrin gene
RLR: RIG-I like receptor
AGS6: Aicardi-Goutières syndrome 6
DSH1: Dyschromatosis Symmetrica Hereditaria 1
*ADARB1*: gene encoding ADAR2
PD-1: programmed cell death 1 protein
PD-L1: programmed cell death 1 ligand 1
